# Towards optimizing single pulse electrical stimulation: High current intensity, short pulse width stimulation most effectively elicits evoked potentials

**DOI:** 10.1016/j.brs.2023.04.023

**Published:** 2023-05-02

**Authors:** Mark A. Hays, Golnoosh Kamali, Mohamad Z. Koubeissi, Sridevi V. Sarma, Nathan E. Crone, Rachel J. Smith, Joon Y. Kang

**Affiliations:** a Department of Biomedical Engineering, Johns Hopkins University, Baltimore, MD, USA; b Johns Hopkins Technology Ventures, Johns Hopkins University, Baltimore, MD, USA; c Department of Neurology, George Washington University, Washington, DC, USA; d Institute for Computational Medicine, Johns Hopkins University, Baltimore, MD, USA; e Department of Neurology, Johns Hopkins University, Baltimore, MD, USA; f Department of Electrical and Computer Engineering, University of Alabama at Birmingham, Birmingham, AL, USA; g Department of Neuroengineering, University of Alabama at Birmingham, Birmingham, AL, USA

**Keywords:** Single pulse electrical stimulation, Cortico-cortical evoked potential, Intracranial EEG, Effective connectivity

## Abstract

**Background:**

While single pulse electrical stimulation (SPES) is increasingly used to study effective connectivity, the effects of varying stimulation parameters on the resulting cortico-cortical evoked potentials (CCEPs) have not been systematically explored.

**Objective:**

We sought to understand the interacting effects of stimulation pulse width, current intensity, and charge on CCEPs through an extensive testing of this parameter space and analysis of several response metrics.

**Methods:**

We conducted SPES in 11 patients undergoing intracranial EEG monitoring using five combinations of current intensity (1.5, 2.0, 3.0, 5.0, and 7.5 mA) and pulse width at each of three charges (0.750, 1.125, and 1.500 μC/phase) to study how CCEP amplitude, distribution, latency, morphology, and stimulus artifact amplitude vary with each parameter.

**Results:**

Stimulations with a greater charge or a greater current intensity and shorter pulse width at a given charge generally resulted in greater CCEP amplitudes and spatial distributions, shorter latencies, and increased waveform correlation. These effects interacted such that stimulations with the lowest charge and highest current intensities resulted in greater response amplitudes and spatial distributions than stimulations with the highest charge and lowest current intensities. Stimulus artifact amplitude increased with charge, but this could be mitigated by using shorter pulse widths.

**Conclusions:**

Our results indicate that individual combinations of current intensity and pulse width, in addition to charge, are important determinants of CCEP magnitude, morphology, and spatial extent. Together, these findings suggest that high current intensity, short pulse width stimulations are optimal SPES settings for eliciting strong and consistent responses while minimizing charge.

## 1. Introduction

The networks of the human brain interact on multiple spatiotem-poral scales, and studying their connectivity is crucial for understanding brain functions. Anatomical connectivity refers to disclosing structural connections via white matter tracts, functional connectivity describes which regions have temporally related neurophysiological activity, and effective connectivity quantifies the influence of one brain region on other regions [1,2]. While methods to quantify connectivity typically involve analysis of passively observed signals from electrophysiological or neuroimaging techniques like EEG or fMRI, single-pulse electrical stimulation (SPES) is an interventional approach increasingly used in intracranial EEG monitoring to quantify the effective connectivity of brain regions [3]. This technique offers an active method for investigating networks in the brain by applying single electrical pulses to one site while measuring the evoked responses in the remaining recording sites. The resulting characteristic responses, known as cortico-cortical evoked potentials (CCEPs), elicited throughout the brain provide information about the directional electrophysiological relationship between the stimulation and response sites [4]. The connections mapped by these responses have been shown to reflect functional networks, such as language [4–7] and motor networks [8], physiological networks corresponding to white matter tracts [6,9,10], and seizure networks, mapping the excitability of epileptogenic regions [11–13].

While CCEPs appear to have multi-faceted utility, there is no standardized procedure or settings for conducting the stimulations. Since electrical stimulation has a very large parameter space, with variables such as polarity, number of phases, frequency, and charge, there is a wide range of settings to conduct SPES [14]. While general principles of electrical stimulation can guide choices for ensuring patient safety, such as keeping a low charge density with charge balanced pulses, the influence of many of these parameters on the resulting CCEPs is not as thoroughly explored. Charge, calculated as the product of current intensity and pulse width, has been most frequently investigated in SPES research but predominately through variation in current intensity with a constant pulse width. Initial CCEP studies using ranges of stimulation intensities showed that CCEP amplitude increases as stimulation intensity is increased [12,13]. This increase and an eventual plateau of CCEP amplitude with increasing current intensity has been further supported by later studies [15–19].

However, CCEP studies that explicitly investigate the effect of varied pulse width have been more limited. Paulk et al. recently showed that increasing pulse width increased the number and spatial spread of responses [19]. Donos et al., who independently varied both the current intensity and pulse width of SPES, found that increasing the charge either by adjusting the current intensity or the pulse width resulted in increased response amplitude, while different current intensity and pulse width combinations with equal charge resulted in similar responses [16]. The authors concluded that charge per phase of the stimulus drives the responses, but further investigation with lower pulse widths and charges per phase more commonly used for CCEPs is needed. Initial CCEP studies favored higher current intensities (up to 12 mA) with shorter pulse widths (0.3 ms) [4,8], compared to the maximum 5 mA current intensity and 5 ms pulse width used by Donos et al. An extensive investigation of the effects of current intensity, pulse width, and charge on several response metrics, such as the spatial distribution and morphology of the evoked potential in addition to the amplitude, using ranges more commonly used for CCEP studies would be beneficial for informing stimulation parameter choice for eliciting CCEPs and enabling comparisons of results across institutions using different settings.

Therefore, in the present study, we sought to investigate the interacting effects of pulse width, current intensity, and charge on responses elicited by SPES through systematic testing of the parameter space and analysis of the responses. We conducted SPES in eleven patients undergoing intracranial EEG monitoring to examine how CCEP amplitude, spatial distribution, latency, waveform similarity, and stimulus artifact amplitude varied for different current intensity and pulse width combinations given the three different constant charge levels and for different pulse widths given the five different constant current intensities. If charge per phase of SPES solely drives the responses, we would expect that parameter combinations with identical charge would result in similar response metrics, while stimulations with greater charge, either by increasing pulse width or current intensity, would result in corresponding changes, such as increased response amplitude and spatial distribution. Knowing how these metrics are influenced by current intensity, pulse width, and charge provides insight into which stimulation parameters are best suited for applying focal stimulations that can elicit strong and robust CCEPs while minimizing stimulus charge.

## 2. Methods

### 2.1. Patients

Eleven drug-resistant epilepsy patients who underwent implantation of intracranial EEG electrodes for seizure localization were included in this study approved by the Johns Hopkins School of Medicine Institutional Review Board. Intracranial EEG was recorded from these patients in the Johns Hopkins Hospital Epilepsy Monitoring Unit from December 2020 through September 2021.

### 2.2. Electrode localization

Stereoelectroencephalography (SEEG) electrodes (AdTech Medical Instruments Corp., Racine, WI, USA) were implanted in each patient except P11 and consisted of 4–14 cylindrical platinum contacts (2.41 mm long, 5 mm spacing). Subdural electrodes (AdTech Medical Instruments Corp., Racine, WI, USA) were also implanted in three of these patients (P3, P9, P11) and consisted of grids or strips of platinum contacts (2.3 mm exposed diameter, 10 mm spacing). The choice of electrode type and coverage was solely driven by clinical reasons decided in the multidisciplinary epilepsy surgery conference. Electrodes were localized by combining presurgical MRI and post-implantation CT using BioImage Suite [20], and electrodes within gray matter were identified using FreeSurfer parcellation and visual verification [21].

### 2.3. Single pulse electrical stimulation

Intracranial EEG recordings were collected with a NeuroPort amplifier (Blackrock Microsystems, Salt Lake City, UT, USA), filtered between 0.3 and 7500 Hz (analog Butterworth antialiasing filters), then digitized at 30 kHz and down-sampled to 1000 Hz. The SPES procedure was conducted using a CereStim R96 (Blackrock Microsystems, Salt Lake City, UT, USA) to apply single pulses of bipolar stimulation to adjacent electrodes at 0.5 Hz. First, full blocks of 40 biphasic pulses with 0.15 ms/phase were delivered at a maximum current intensity (5–10 mA, median 10 mA) that elicited visually consistent evoked responses without causing after-discharges. Following stimulation of all possible electrode pairs within gray matter, three to five stimulation sites were selected in each patient for variable pulse width and current intensity blocks. Due to limited time, these selections were based on visual inspection of the average responses to the initial full blocks, choosing sites where stimulation resulted in many, high amplitude evoked responses. The results from the initial full blocks were otherwise not used in this study.

**Fig. 1.**
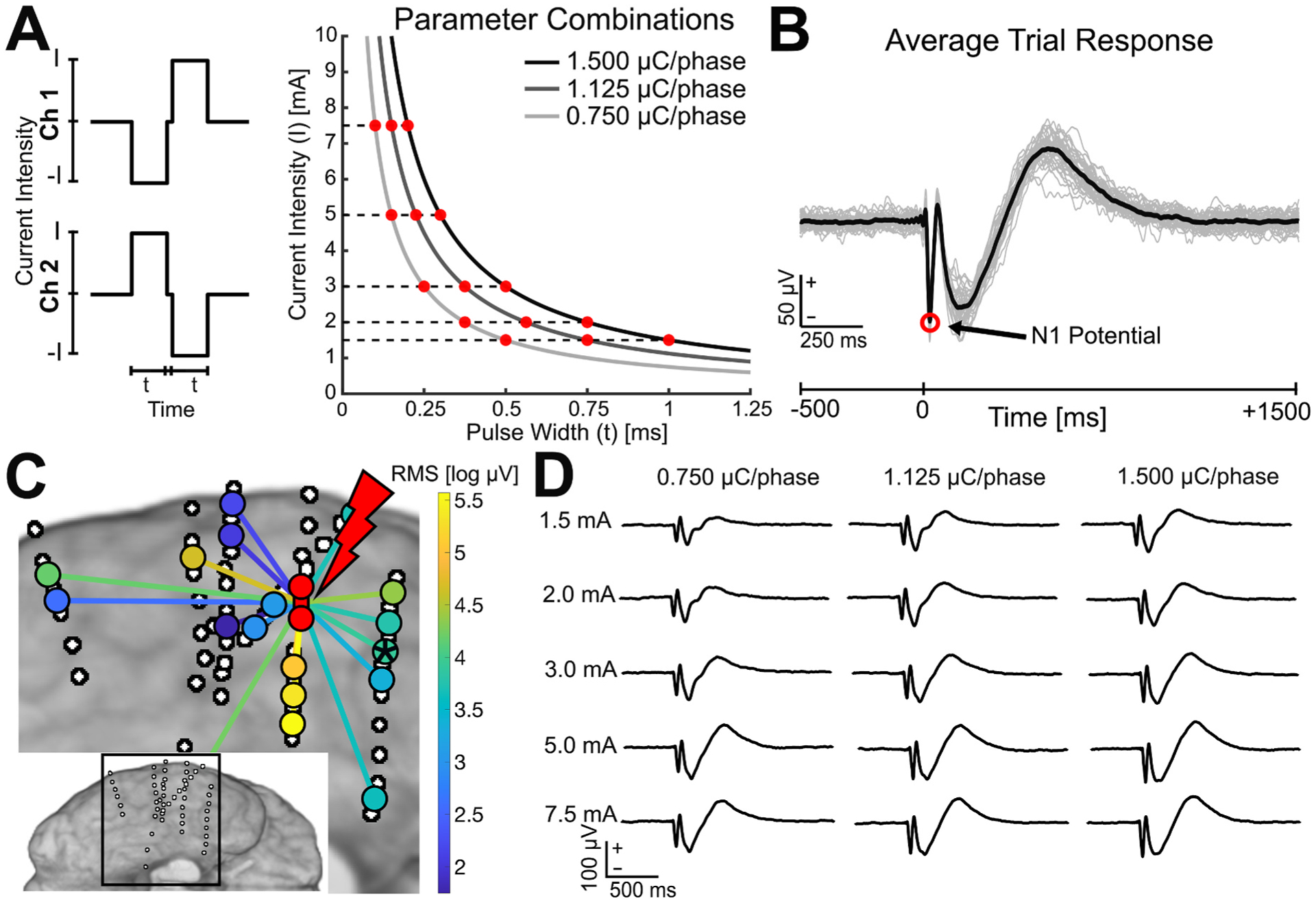
Single pulse electrical stimulation (SPES) procedure. (**A**) SPES was applied using bipolar stimulation of adjacent electrodes with 40 biphasic pulses applied at 0.5 Hz. At each site, 15 blocks were conducted using five current intensities (1.5, 2.0, 3.0, 5.0, and 7.5 mA) and three varied pulse widths so that each block using the same current intensity had a different total charge (0.750, 1.125, 1.500 μC/phase). Individual current intensity and pulse width combinations are shown on the isocharge curves. (**B**) Trial-averaged evoked responses in each bipolar montage channel were computed to obtain cortico-cortical evoked potentials (CCEPs) in response to each stimulation site and each of the 15 stimulation parameter combinations. The amplitude and latency of the N1 potential was identified in each CCEP from 10 to 50 ms post-stimulus. (**C**) For each block, responses with an N1 potential greater than six standard deviations of the pre-stimulus baseline (−500 to −10 ms) were classified as significant. Response amplitude was additionally quantified by the root-mean-squared (RMS) of the signal from 10 to 100 ms, as a more general measure of the early response magnitude. (**D**) Example CCEPs recorded at the channel denoted with an asterisk in **C** in response to each of the stimulation parameter conditions tested.

For the selected sites, three blocks of SPES with 40 biphasic pulses were conducted at each of five current intensities (1.5, 2.0, 3.0, 5.0, and 7.5 mA) using varied pulse widths so that each block used a different total charge (0.750, 1.125, and 1.500 μC/phase). This resulted in 15 total blocks for each stimulation site (Fig. 1A, Table 1). In this way, the effect of varied pulse width could be examined per each of five different current intensities, and the effect of using different current intensity and pulse width combinations while keeping charge constant could be examined for three different charge levels. With an electrode surface area of 0.081 cm^2^ (SEEG) or 0.042 cm^2^ (subdural), the charge densities were 9.26, 13.89, and 18.52 μC/cm^2^/phase for SEEG and 18.05, 27.08, and 36.10 μC/cm^2^/phase for subdural grids and strips, which were well within safety limits [22]. The CereStim stimulator imposed a minimum interphase duration of 0.053 ms between each phase of the biphasic pulse. Additionally, the 1 μs precision of the pulse width setting required that the pulse width for 2.0 mA at 1.125 μC/phase be rounded from 0.5625 ms to 0.563 ms.

Electrode channels were re-referenced to a bipolar montage after rejection of channels with excessive noise on visual inspection. Stimulus artifacts were removed by replacing the data from −5 to 10 ms around the stimulus with reversed, tapered copies of the data before and after this period [23]. Next, signals were low pass filtered at 50 Hz and segmented by trial into windows from −500 to 1500 ms time-locked relative to each stimulation. Data was processed and analyzed using custom Matlab scripts (R2021a, MathWorks, Natick, MA, USA).

### 2.4. Evoked potential response metrics

The windowed signals were baseline-centered ( −500 to −10 ms baseline) and averaged over the 40 trials for each block to obtain average responses of each channel to each current intensity and pulse width combination used at each stimulation site. Significant CCEPs were identified from these average responses using the amplitude of the N1 potential, a characteristic early (10–50 ms latency) negative potential that is thought to represent the direct connectivity between stimulation and response sites [3,4,8]. The amplitude and latency of the N1 potential for each response was identified using peak detection within 10–50 ms post-stimulus. The absolute value of a response’s N1 amplitude was normalized by the standard deviation of the baseline to obtain a Z-score and was considered significant if the Z-score was greater than six standard deviations [5,15]. If a stimulation-response pair had a significant response to at least one of the combinations of current intensity and pulse width, its responses to all the variable pulse width and current intensity blocks were included for further analysis.

While the N1 potential was used to determine whether a CCEP was significant, we used the root mean squared (RMS) of the early response from 10 to 100 ms post-stimulus to quantify the response amplitudes for analysis. RMS of the early response is a robust metric of CCEP connectivity increasingly used to quantify the strength of responses elicited from SPES [16,24–26]. RMS was used rather than N1 amplitude because it is a more general measure that can quantify responses when there is no distinct N1 potential. This allows for more appropriate paired comparisons between stimulation parameters since the same stimulation-response pair may have a significant N1 potential in response to some current intensity and pulse width combinations but not to others that may not have been strong enough. The 10–100 ms window was chosen to capture the full deflection of the N1 potential while leaving out the slower N2 potential, typically occurring 100–300 ms post-stimulus, which presumably results from indirect connectivity [3, 4].

We quantified the spatial distribution of responses using the number of significant responses and the distance of those responses from the stimulation site. First, the proportion of response sites with a significant response (Z-score >6) was calculated for each stimulation site and stimulation parameter combination. Then, using the Euclidean distance between the stimulating electrodes and the response electrodes obtained by the electrode localization, the distance of the significant responses was calculated for each stimulation site and stimulation parameter combination.

### 2.5. Stimulus artifact amplitude quantification

To examine how different stimulation parameters influenced the area activated by stimulation, we relied on the stimulus artifacts observed at electrodes surrounding the stimulated site. While stimulus artifacts are often removed before analysis, the artifact amplitude can be used to quantify the strength of activation of the tissue due to volume conduction during SPES, representing the electric field induced by the stimulus [27]. The maximum absolute value of the voltage from −2 to 5 ms in each channel was averaged over each of the 40 trials to obtain an artifact amplitude for each stimulation-response pair for each current intensity and pulse width combination. We used the amplitudes of stimulus artifacts within 30 mm of the stimulation site as a metric of how strongly the region surrounding the stimulation site was activated by the stimulation. Comparing the stimulus artifact amplitudes produced by each current intensity and pulse width combination facilitated the assessment of the spatial selectivity of the stimulation parameters, since stimulations that are less focal will produce larger artifacts within this region. We chose the 30 mm distance since previous SPES studies have estimated that contacts up to 20–30 mm from stimulation are within the volume conduction zone [27,28].

## 3. Statistical analysis

### 3.1. Response metric comparisons

We compared several metrics to analyze how changing the stimulation parameters of current intensity, pulse width, and charge affects CCEPs: response amplitude (RMS from 10 to 100 ms), spatial distribution (proportion and distance of significant responses), N1 latency, and stimulus artifact amplitude. For each metric, we performed pairwise comparisons between each current intensity and pulse width combination using data from all patients, paired by either the same stimulation-response pair (for response amplitude, N1 latency, and artifact amplitude) or by the same stimulation site (for proportion and distance of significant responses). Statistical differences between each combination were calculated by Wilcoxon signed-rank tests, and significance was determined using Bonferroni corrected *P* < 0.05. Response amplitude was compared for all stimulation-response pairs that had a significant response in at least one of the 15 parameter combinations. For N1 latency, only stimulation-response pairs that had a significant response to all combinations being compared were included to ensure that the latency is quantifying the timing of an identified N1 potential. Additionally, to compare waveform morphologies, the Pearson correlation between the average time series of the same stimulation-response channel pair was computed for each parameter combination.

Prior to comparisons, the response amplitudes and stimulus artifact amplitudes were log-transformed to normalize the distributions. While the distributions were still significantly non-normal, the normality was improved (Kolmogorov-Smirnov tests, RMS before log-transformation *D* = 0.297, *P* < 0.001, after *D* = 0.027, *P* < 0.001; artifact amplitudes before log-transformation *D* = 0.363, *P* < 0.001, after *D* = 0.071, *P* < 0.001). The log-transformation means that interpretations of the differences in log amplitudes between stimulation parameters translate to multiplicative differences in the raw voltages rather than additive differences.

### 3.2. Linear mixed effect models

Since CCEP amplitude is widely used to quantify the responses, we further investigated the effects of stimulation parameters on the response amplitude through linear mixed effects models. A full model fitting the log-transformed response amplitudes to fixed effects of current intensity, pulse width, and charge (the interaction effect of current intensity and pulse width) and a random effect of stimulation-response pair was compared to reduced null models that lack each fixed effect to determine the significance. We fit the reduced models without each fixed effect to the data and used likelihood ratio tests (LRT) to compare these to the full model. Statistical significance was calculated using the *P*-values from the LRT statistics on chi-squared distributions with degrees of freedom equal to the difference in degrees of freedom between full and reduced models.

## 4. Results

Eleven patients (5 male, 6 female, median age 33, range 11–54) were included in this study (Table 2). We chose a median of 4 sites (range 3–5) in each patient for stimulation with variable current intensity and pulse width combinations. Each site was stimulated using the same 15 different combinations of current intensity and pulse width, except for P1 in which only the 5 different current intensity and pulse width combinations at 0.750 μC/phase were performed and for P4 in which the 5.0 and 7.5 mA stimulations at 1.500 μC/phase were not performed at one of the sites. A median of 109 stimulation-response pairs (range 82–198) were classified as significant (N1 Z-score in response to at least one parameter combination was greater than 6) in each patient across all stimulations.

### 4.1. Response amplitude

**Fig. 2.**
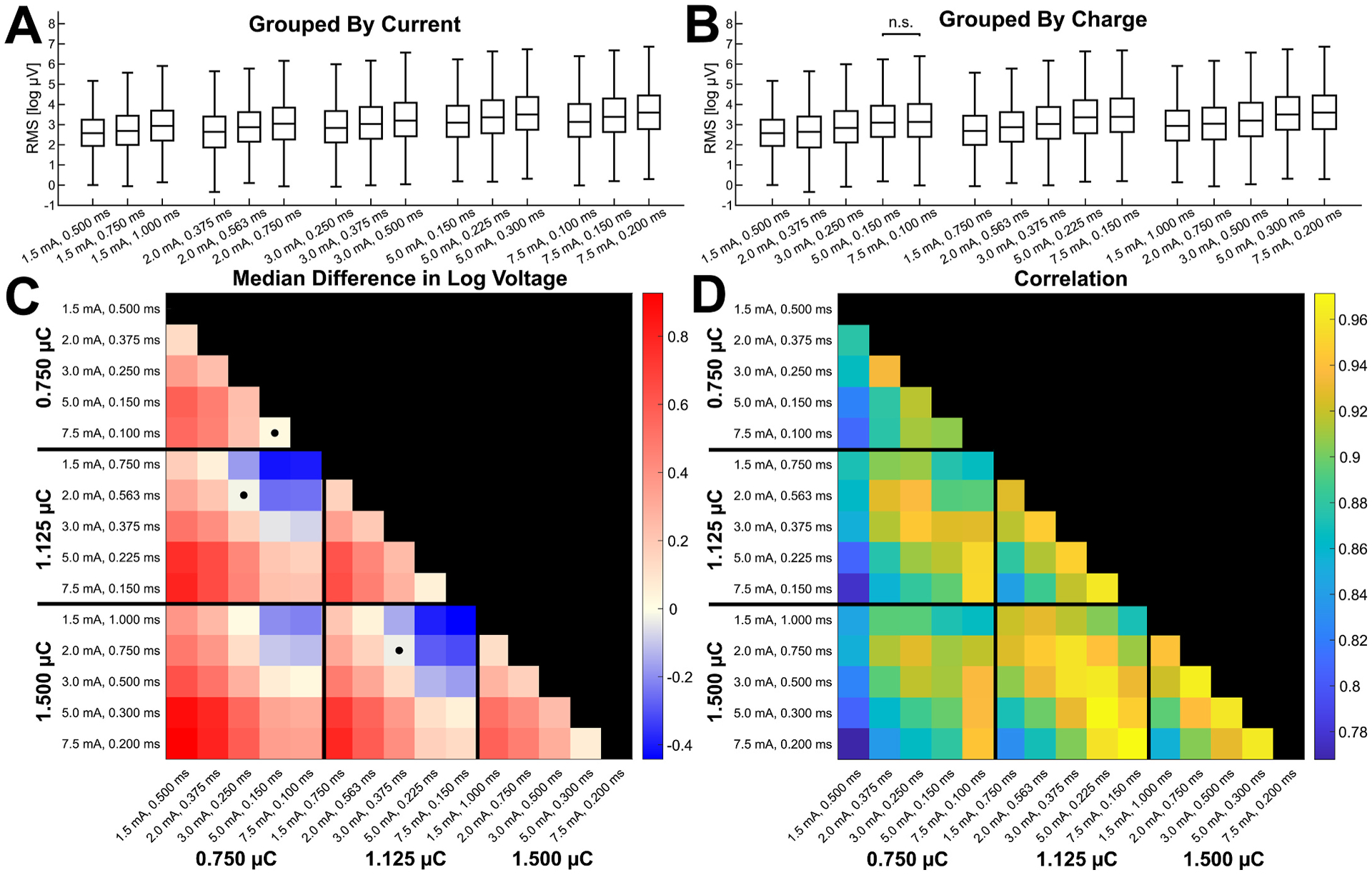
Pairwise comparisons of response magnitude between varied current intensity and pulse width combinations. Boxplots of response amplitudes at each parameter combination are shown spatially grouped by current intensity in **A** and spatially grouped by charge in **B**. Wilcoxon signed-rank tests between pairwise comparisons within each current intensity and within each charge level were significant (*P* < 0.05, Bonferroni corrected) except where labeled non-significant (n.s.). The median differences in response amplitude across each stimulation-response pair for all possible pairwise comparisons of the 15 parameter combinations are shown in **C**. The value of each square represents the condition on the y-axis minus the condition on the x-axis, and the matrix is colored so that a greater value for the condition on the y-axis is colored red and a greater value for the condition on the x-axis is colored blue. The values are dimensionless since they quantify the difference in log-scaled voltages. Squares in **C** marked with a dot represent comparisons with non-significant differences (Wilcoxon signed-rank tests, *P* > 0.05, Bonferroni corrected). While stimulations with a greater charge generally resulted in a greater amplitude than those with a lower charge, stimulations with a greater charge but a lower current intensity (1.5–2.0 mA) sometimes resulted in lower magnitude responses compared to those from stimulations with a lower charge but a higher current intensity (5.0–7.5 mA). The Spearman’s correlation coefficient for the response amplitude between each combination of current intensity and pulse width is shown in **D**. The correlation generally increased with both the magnitude and the similarity of the current intensities and overall charges of the stimulation parameters being compared.

Fig. 2A shows the distributions of the response amplitudes, as quantified by the RMS from 10 to 100 ms post-stimulus, in response to each stimulation parameter combination grouped by constant current intensity. Fig. 2B shows the same distributions grouped by constant charge. The paired differences in response amplitude across varied pulse widths at a constant current intensity were significant for every pairwise comparison at each current intensity (Wilcoxon signed-rank tests, *P* < 0.05) (Fig. 2A). Stimulations with longer pulse widths while keeping current intensity constant resulted in significantly greater response amplitudes, as expected from the increase in charge. However, nearly all the combinations of current intensity and pulse width with the same overall charge also had significant paired differences in response amplitude (Fig. 2B). Notably, constant charge combinations that had greater current intensities and shorter pulse widths resulted in responses with significantly greater amplitudes than combinations that had lower current intensities and longer pulse widths (Wilcoxon signed-rank tests, *P* < 0.05).

When looking at all possible pairwise comparisons between the 15 combinations of current intensity and pulse width, an interesting pattern emerged. We computed the median paired difference in amplitude across all significant connections to obtain one value for each pairwise comparison shown in Fig. 2C, where red indicates the amplitudes in response to the condition on the y-axis were greater while blue indicates those in response to the condition on the x-axis were greater. While most stimulations with a greater charge resulted in a greater amplitude when compared to stimulations with a lower charge, this was not always the case. Specifically, stimulations with a greater charge but a low current intensity and long pulse width sometimes resulted in lower magnitude responses compared to those from stimulations with a lower charge but a high current intensity and short pulse width. This can be seen by the blue colored squares in Fig. 2C where stimulations with a greater charge but the lowest current intensities of 1.5 or 2.0 mA were compared with stimulations with a lower charge but the greatest current intensities of 5.0 or 7.5 mA.

In addition to the paired comparisons of amplitude, the Spearman correlation of response amplitudes between each combination of current intensity and pulse width was computed to examine how similar the response distributions were (Fig. 2D). The correlation was greater between stimulations that have greater current intensities and greater overall charge. When comparing parameter combinations that have different charges, the correlation tends to be greatest between combinations that have similar current intensities.

### 4.2. Spatial distribution

**Fig. 3.**
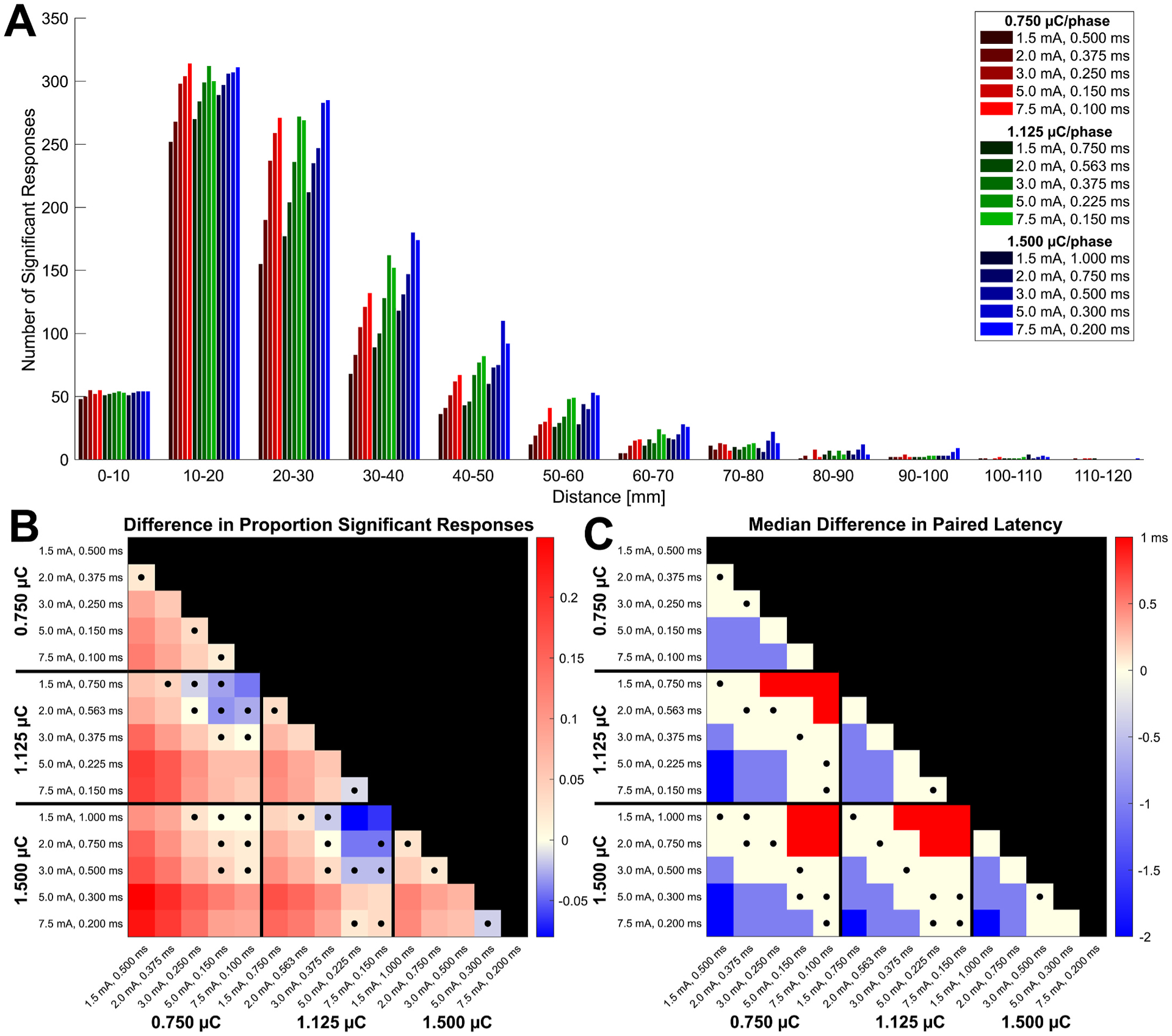
Comparisons of spatial distribution and latency of responses. The histogram in **A** shows the distributions of the distances of significant responses observed for each stimulation condition across all patients. The median differences in the proportion of responses that were significant across each stimulation for all possible pairwise comparisons of the 15 current intensity and pulse width combinations are shown in **B**. The median differences in N1 response latency across each stimulation-response pair for all possible pairwise comparisons of the 15 current intensity and pulse width combinations are shown in **C**. In both matrices, the value of each square represents the condition on the y-axis minus the condition on the x-axis, and the matrix is colored so that a greater value for the condition on the y-axis is colored red and a greater value for the condition on the x-axis is colored blue. Squares marked with a dot represent comparisons with non-significant Wilcoxon signed-rank tests (*P* > 0.05, Bonferroni corrected). Together, stimulations with a greater charge or current intensity generally resulted in a greater proportion of significant responses and shorter latencies, but stimulations with a high charge and the lowest current intensities sometimes had a lower proportion of significant responses and longer latencies compared to stimulations with a lower charge and highest current intensities.

The distribution of the number of significant responses observed within each 10 mm interval from the stimulation site is shown for each parameter condition in Fig. 3A. While differences in distances of significant responses were largely non-significant (Supplementary Figure 1), Fig. 3A shows the trend that stimulations with the largest current intensities and the largest charges tended to have an increased number of longer distance connections. The median pairwise differences in the proportion of responses that were significant for each stimulation are shown in Fig. 3B. When comparing between stimulations that used the same charge, stimulations with a higher current intensity and shorter pulse width tended to result in significantly greater proportions of significant responses (Wilcoxon signed-rank tests, *P* < 0.05). While stimulations with a greater charge generally resulted in a significantly greater proportion of significant responses, stimulations with a low current intensity and long pulse width sometimes had a lower proportion of significant responses compared to stimulations with a greater current intensity and shorter pulse width but lower charge. This is shown by the blue colored squares in Fig. 3B, and it was only significant when comparing the combinations with the greatest difference in current intensities and the least difference in overall charges (1.5 and 2.0 mA at 1.500 μC compared to 5.0 and 7.5 mA at 1.125 μC).

### 4.3. Latency

We next compared the N1 latencies of stimulation-response pairs that had significant N1 potentials in response to both combinations of current intensity and pulse width being compared. The median paired differences in the latencies between each parameter combination are shown in Fig. 3C, where red indicates a longer latency and blue indicates a shorter latency for the condition on the y-axis. Example waveforms from a single representative stimulation-response pair are additionally shown in Supplementary Figure 2 to help visualize latency differences. While the median difference between conditions was often 0 ms, the Wilcoxon signed-rank tests between combinations with more extreme differences in charge and current intensity were significant (*P* < 0.05). For comparisons between constant charge conditions, stimulations with greater current intensities generally resulted in shorter N1 latencies than did the stimulations with lower current intensities, although the median paired difference was usually only 1 ms in magnitude (Fig. 3C). Stimulations with greater charge also generally resulted in shorter (median paired difference of 2 ms at most) or equivalent latencies, though stimulations with the lowest current intensity and longest pulse width resulted in longer N1 latencies (median paired differences of 1 ms) than stimulations with a lower charge but the greatest current intensities and shortest pulse widths, shown by the red squares in Fig. 3C (1.5 mA at 1.125 and 1.500 μC compared to 5.0 or 7.5 mA at 0.750 and 1.125 μC). Comparisons of the differences in latency in proportion to the average latency of a channel’s response are shown in Supplementary Figure 3 along with the correlation between the latency difference and the average latency. These indicate that there may be a trend for differences in N1 latency to occur in proportion to the magnitude of the N1 latency of the response observed at that site.

### 4.4. Waveform similarity

**Fig. 4.**
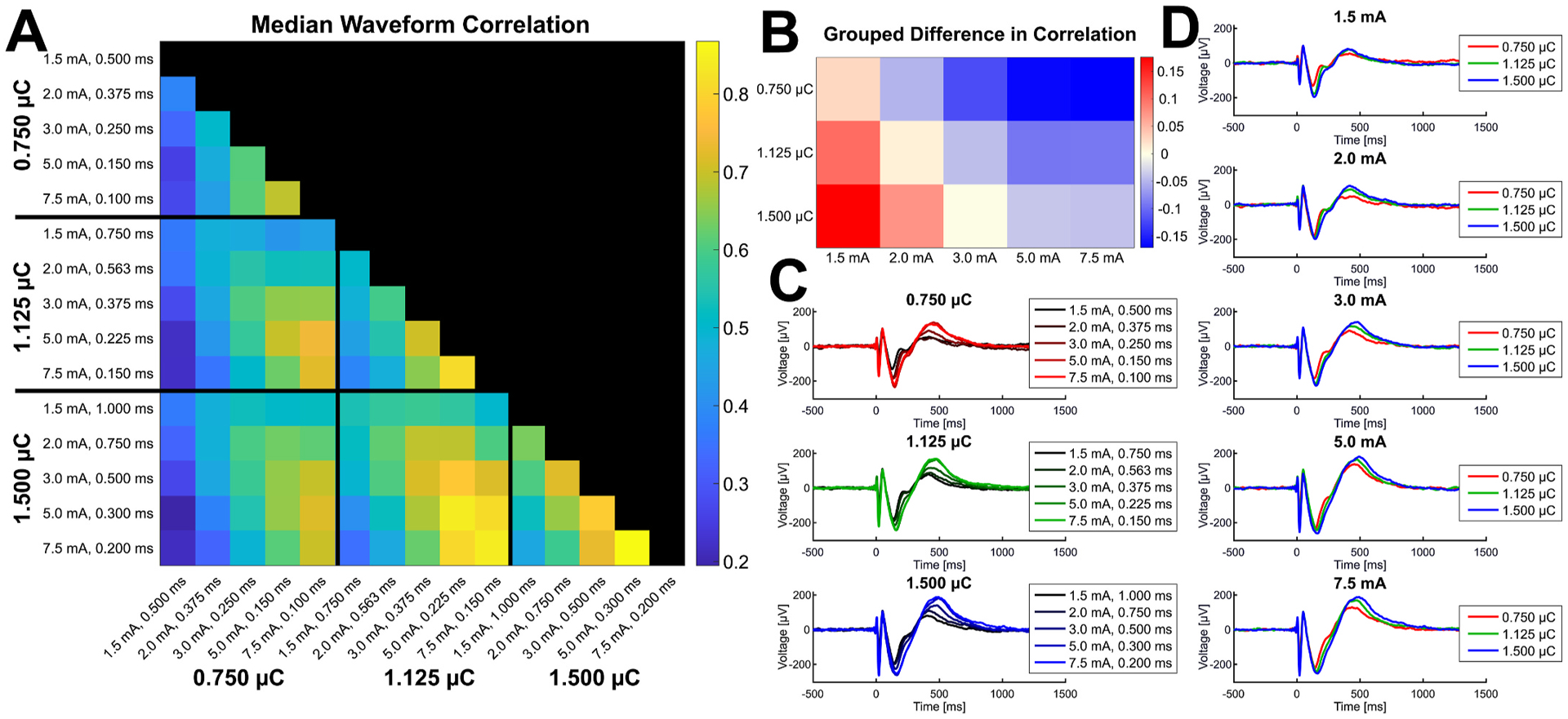
Waveform Correlation. The Pearson’s correlation coefficient between the average response time series of the same stimulation-response pair in each combination of current intensity and pulse width was calculated, and the median coefficient across all stimulation-response pairs is shown in **A** for each pairwise comparison. The median correlation of all conditions that used the same current intensity and all conditions that used the same charge was calculated for each stimulation response pair, and the difference in the medians across all stimulation-response pairs between each constant current intensity and constant charge grouping is shown in **B**. Each square represents the condition on the y-axis minus the condition on the x-axis, and the matrix is colored so that a greater value for the condition on the y-axis is colored red and a greater value for the condition on the x-axis is colored blue. Example waveforms of the average times series of one stimulation-response pair in each stimulation condition are shown grouped by charge (**C**) and current intensity (**D**) to visualize the trends. Overall, waveform correlation increased with current intensity and charge, and above 3 mA the correlation between waveforms in response to the same current intensity but different pulse widths was greater than the correlation between waveforms in response to the same charge but different current intensities and pulse widths.

The average response waveform elicited by one current intensity and pulse width combination was compared to that of the same stimulation-response pair under each other condition by computing the Pearson’s correlation coefficient between each pair of average response time series. The median Pearson’s correlation coefficients are shown in Fig. 4A for each pairwise comparison of parameter conditions. Correlation was greatest between waveforms in response to the greatest current intensities and charges. We next examined whether stimulations using the same current intensity with different charges or stimulations using the same charge with different current intensities resulted in more highly correlated waveforms. For each stimulation-response pair, the median correlation coefficient within each current intensity was compared to the median correlation coefficient within each charge level. The median difference in these values across all stimulation-response pairs is shown in Fig. 4B, with each colored square representing the condition on the y-axis minus the condition on the x-axis, and all Wilcoxon signed-rank tests were significant (*P* < 0.05). The correlation between the waveforms in response to stimulations with the same current intensity but different pulse widths increased with current intensity, and by 3.0–5.0 mA it was greater than the correlations of waveforms in response to the same charge level for each charge level used. This trend is also visualized by example waveforms from a single representative stimulation-response pair shown in Fig. 4C and D.

### 4.5. Stimulus artifact amplitude

**Fig. 5.**
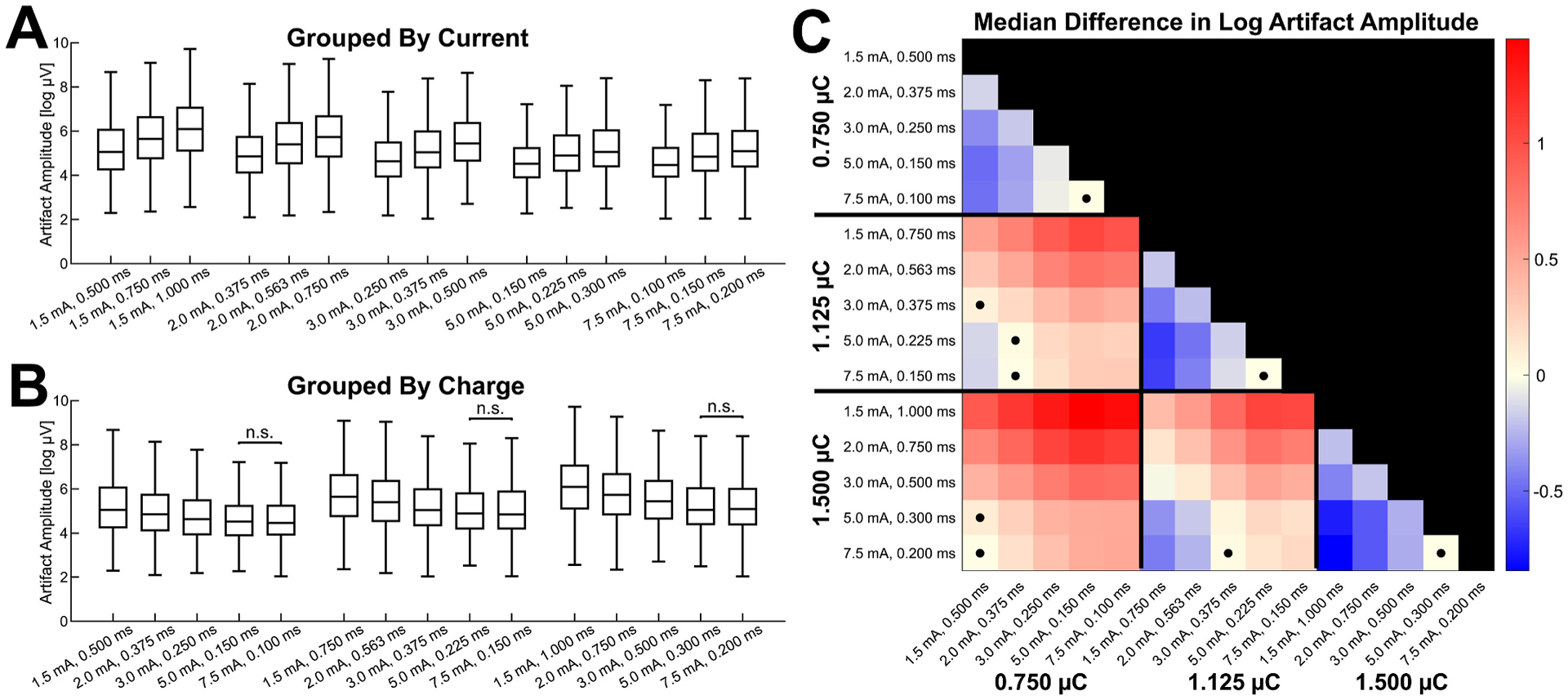
Pairwise Comparisons of Artifact Amplitude as a Measure of Focal Activation. Boxplots of stimulus artifact amplitudes at each current intensity and pulse width combination are shown spatially grouped by current intensity in **A** and spatially grouped by charge level in **B**. Wilcoxon signed-rank tests between pairwise comparisons within each current intensity and within each charge level were significant (*P* < 0.05, Bonferroni corrected) except where labeled non-significant (n.s.). The median differences in stimulus artifact amplitude across each stimulation-response pair for all possible pairwise comparisons of the 15 current intensity and pulse width combinations are shown in **C**. The value of each square represents the condition on the y-axis minus the condition on the x-axis, and the matrix is colored so that a greater value for the condition on the y-axis is colored red and a greater value for the condition on the x-axis is colored blue. The values are dimensionless since they quantify the difference in log-scaled voltages. Squares in **C** marked with a dot represent comparisons with non-significant Wilcoxon signed-rank tests (*P* > 0.05, Bonferroni corrected). Stimulations using a greater charge generally resulted in greater stimulus artifact amplitudes, and for a constant charge, stimulations with shorter pulse widths and higher current intensities resulted in lower artifact amplitudes.

The pairwise comparisons of the amplitude of stimulus artifacts from sites within 30 mm of each stimulating electrode pair are shown in Fig. 5. The distributions of the artifact amplitudes for each stimulation parameter combination are shown grouped by constant current intensity in Fig. 5A and grouped by constant charge in Fig. 5B–aid visual comparisons of the results, and the median paired differences in artifact amplitude for all pairwise comparisons are shown in the matrix in Fig. 5C. Stimulations using longer pulse widths at a constant current intensity resulted in significantly greater artifact amplitudes, for each current intensity (Wilcoxon signed-rank tests, *P* < 0.05) (Fig. 5A). However, stimulations with shorter pulse widths while keeping charge constant resulted in lower artifact amplitudes (Fig. 5B). Only the comparisons between 5 mA and 7.5 mA at each charge level were non-significant. Comparisons across different charge levels showed that a greater charge generally resulted in greater amplitude stimulus artifacts, except when there were more extreme differences in pulse width or current intensity. This is visualized in Fig. 5C by the comparisons between different charge levels being mostly red, except the few squares of blue at the extremes where the pulse width of the greater charge is much shorter than the pulse width of the combination with the lower charge (5.0 and 7.5 at 1.125 C compared to 1.5 mA at 0.750 μC, or 5.0 and 7.5 at 1.500 μC compared to 1.5 and 2.0 mA at 1.125 μC).

### 4.6. Mixed effects model

The response amplitudes of all stimulation-response pairs with at least one significant response across all parameter conditions were fit with linear mixed effect models to further examine the interacting effects of current intensity, pulse width, and charge. The full model with fixed effects of current intensity, pulse width, charge was significantly better fit than each reduced model (LRT, *P* < 0.0001, Supplementary Table 1). Most notably, the full model was significantly better fit than the model with charge as the only fixed effect (*LRT*(2) = 5416.702, *P* < 0.0001), indicating that the particular combination of current intensity and pulse width used for a given charge does also affect the expected response amplitude. The coefficients for the fixed effects in the full model are shown in Table 3, and each effect was significant. The coefficients for current intensity and pulse width cannot be interpreted directly without the influence of the interaction term representing charge. However, since the coefficient for current intensity is positive and the coefficient for pulse width is negative, this indicates that when charge is held constant, stimulations with a greater current intensity and a lower pulse width would result in greater expected response amplitudes, matching our observations described above.

This fit model may be used to predict which other combinations of current intensity and pulse width would be expected to result in similar response amplitudes. Given an initial current intensity and pulse width, a different stimulation using a lower current intensity must increase the pulse width greater than what would be necessary to keep charge constant to get similar responses according to this model. To see how well this model could capture the observed findings, we calculated predicted voltage values for each combination of current intensity and pulse width we used, representing the predicted average response amplitudes across all stimulation-response pairs, for each combination of current intensity and pulse width. The differences in those values between each pairwise comparison of stimulation parameters were then compared to the differences in observed values, and the trends and magnitudes were well aligned (Supplementary Figure 4). Although this is a simplistic model that does not necessarily reflect any neurophysiological principles that relate current intensity, pulse width, and charge to response voltage, it can still capture the general effect that is observed: while stimulation charge largely drives the amplitude of elicited CCEPs, the particular current intensity and pulse width combination is also important.

## 5. Discussion

It is important to understand how stimulation parameter choices for SPES affect the ability to elicit robust CCEPs. We conducted SPES in eleven patients using five combinations of current intensity and pulse width at each of three different charge levels to better elucidate how the interacting effects of charge, current intensity, and pulse width influence several relevant CCEP metrics: amplitude, spatial distribution, latency, and morphology. We found that stimulations with a greater charge or stimulations with a greater current intensity and shorter pulse width at a given charge generally resulted in greater response amplitudes, greater proportions of significant responses, shorter latencies, and increased correlation of waveforms. These effects interacted such that low charge stimulations with the highest current intensities (5.0–7.5 mA) resulted in more favorable results (greater response amplitudes and distributions, shorter latencies, more consistent waveforms) than did stimulations with a greater charge but the lowest current intensities (1.5–2.0 mA). Further, while the stimulus artifact amplitude, used here to quantify the strength of activation of surrounding tissue, generally increased with charge, this effect could be mitigated by using stimulations with a high current intensity and short pulse width. The effects of the stimulation parameters on response magnitude were further examined using linear mixed effect models to show that charge, current intensity, and pulse width were all significant factors in determining the response amplitude, producing a model useful for predicting the average expected response amplitude when changing stimulation parameters.

Our results suggest that while using stimulations with greater charge may be generally favorable for reliably producing evoked responses, one could instead use a lower charge with a higher current intensity and shorter pulse width to maximize patient safety through reduced charge density while retaining the ability to elicit strong, numerous CCEPs. This indicates that the current intensity of the stimulation is more influential on the evoked response amplitude than the pulse width is. While this observation differs from the conclusion drawn in Donos et al., 2016 [16] that the charge per phase of the stimulus, regardless of the current intensity and pulse width, drives response amplitude, Donos et al. did also report that there was greater correlation between the charge per phase and response amplitude when current intensity was varied compared to when pulse width was varied. We believe our findings to be a nuance of those in Donos et al., 2016 [16] due to differences in stimulation parameters ranges tested, rather than a conflicting result. While we chose current intensities and pulse widths that spanned typical ranges used in CCEP studies (1.5–7.5 mA and 0.1–1 ms), the charges per phase were relatively low, ranging from 0.750 to 1.500 μC/phase. Charge was intentionally kept low to test what stimulation parameters could result in robust responses while minimizing the charge delivered, but this may account for differences between our observed results and those observed in Donos et al., 2016 [16], which spanned a range of 0.75–15.0 μC/phase. Since response amplitudes tend to plateau with greater intensity stimulations [13,17,18], it is possible that at greater charges, the stimulation is already maximally activating possible connections and saturating the responses, leading to less observed differences when the combination of current intensity and pulse width is adjusted. A further study performing a similar experiment with a larger range in charge may be needed to investigate whether the pattern we observed is maintained for greater charges.

One possible explanation for why we observed different current intensity and pulse width combinations at the same charge to affect response magnitude may be gathered from previous research in neural stimulation that uses a strength-duration relationship to describe the threshold charge *Q*_*th*_ required to generate an action potential using a stimulation with pulse width *t*. This is defined by *Q*_*th*_ = *b*(*t* + *c*), where the rheobase current *b* is the current required to elicit a response given an infinitely long pulse width, and the chronaxie *c* is the pulse width required to elicit a response using a current that is double the rheobase current [29–31]. Since the charge threshold increases linearly with pulse width, stimulations using a longer pulse width would require an overall greater charge to trigger an action potential. While this describes the production of a binary action potential response, the relationship seems to fit the trend in our observed results for responses with a continuous magnitude. If the charge threshold to elicit the same magnitude response increased with pulse width, this would correspond with why the particular combination of current intensity and pulse width affected the response magnitude in addition to the charge per phase. Combinations with a long pulse width resulted in smaller response amplitudes than did combinations of equivalent charge with a shorter pulse width, perhaps because the charge threshold to elicit that same response was greater due to the longer pulse width.

Rather than focusing solely on the CCEP amplitude, our study is unique in investigating how varying current intensity and pulse width interact to affect several other measures of responses to SPES, since maximizing response magnitude may not be the only factor in deciding optimal stimulation settings. When examining the spatial distribution of responses, we observed that the distance and proportion of significant connections both generally increased with charge, which fits with previous findings showing that greater charge levels activated more connections, usually more distant [19,32]. While differences in N1 latency across parameter conditions were relatively small (median of 2 ms at most), many were still significant. The most notable differences were that high current intensity, short pulse width stimulations resulted in shorter latencies than did low current intensity, long pulse width stimulations. The relatively long latency (10–50 ms) and blunt peak of the N1 potential is hypothesized to result from jittered activation of the local area surrounding the stimulus, resulting in a jitter of synaptic activity in the stimulated cortex as well as at connected sites where CCEPs are observed [3,4,8]. If this is the generator mechanism, our results may be explained by the higher current intensity activating the surrounding area quickly, leading to a fast, high magnitude peak in the response, while stimulations with a lower current intensity and long pulse width result in a more spread-out jitter of activation leading to a slower, lower magnitude response peak. It is possible that latency could have been affected by other factors, since the latency of the N1 potential is positively correlated with both distance and the number of white matter pathways between stimulation and recording sites [6,32,33]. However, since the latency comparisons in this study were paired by the same stimulation and response site to best control for these effects, we do not believe these factors to account for or have interfered in the observed differences.

Compared to the narrow focus of the N1 potential that reflects the direct connectivity, quantifying the overall morphology of the response waveform captures variability in additional later components of the CCEP, such as the N2 potential, that may arise from indirectly excited circuits at the response sites or direct slow multisynaptic activities through subcortical pathways [3,4]. Previous studies have shown that waveform morphology does vary with current intensity [13,15], and the similarity of CCEP waveforms increases with current intensity and eventually plateaus [17–19]. We similarly observed the waveform morphology to be more highly correlated between stimulations with greater charges and current intensities. This increased stability of the waveform morphology at greater current intensities and charges could be indicative of maximal activation of the surrounding region, recruiting additional ensembles responsible for the later components of the CCEP [17]. We also observed that above 3.0–5.0 mA, there was generally greater correlation between waveforms in response to stimulations with the same current intensity but different pulse widths than to stimulations with the same charge but different current intensity and pulse width combinations. This suggests that robust consistent waveforms can be elicited by using a constant, high current intensity rather than simply maximizing stimulation charge.

In stimulation applications, varying charge through either current intensity or pulse width can affect how spatially selective the region around the stimulated electrodes is activated [14]. Since the electrical current required to excite a region past its threshold increases proportionally to the inverse square of the distance from stimulation site, current intensity can determine the maximum area excited by stimulation, with lower current intensities being more focal [34]. Similarly, shorter pulse width stimulations can produce more spatially selective activation in applications such as deep-brain stimulation and peripheral nerve stimulation [35,36]. We observed similar effects in SPES by using the stimulus artifact amplitude as a measure of the strength of activation of surrounding tissue during stimulation, finding that stimulus artifact amplitude generally increased with charge. Prime et al. similarly observed that stimulus artifact amplitude increased with charge by varying stimulation current intensity for a fixed pulse width [27]. However, we also observed that stimulations with the same charge but a greater pulse width and lower current intensity resulted in greater stimulus artifact amplitudes, indicating that pulse width may affect spatial selectivity of the stimulation more than current intensity does. For a given charge level, favoring a high current intensity and short pulse width may reduce stimulus artifact amplitude, reflecting a more focal stimulation and ensuring that the evoked potentials reflect effective connectivity from the stimulation site, rather than the surrounding region affected by volume conduction [27,28].

One limitation of this study is the limited spatial sampling of the stimulation sites. Because of the amount of time required to conduct all the stimulation blocks with each set of current intensity and pulse width parameters, only 3 to 5 stimulation sites were chosen in each patient due to time constraints. As a result, we did not have a sample size large enough to directly compare observed results within and between connections of different anatomical regions, and the results we did observe may have been influenced by the underlying structural and functional connections of the selected stimulation sites [9,10]. Differences based on tissue type have also been reported, such as increased connection probability from white matter stimulations [32], nonlinear effects of stimulation distance and orientation with respect to the gray-white matter boundary [19], and decreased CCEP amplitude from response sites in white matter [17]. However, others have observed no discernible differences in stimulus artifact and early response amplitudes between gray and white matter [27]. In this study, due to the limited spatial sampling, we relied on a homogeneous assumption by grouping all connections from all patients together, and we controlled for differences by ensuring that responses to different stimulation parameters were only compared to other responses observed between the same stimulation and response sites.

Lastly, we chose to focus on charge, current intensity, and pulse width and did not investigate other stimulation parameters such as polarity or number of phases. While bipolar stimulation is typically used in SPES studies since it activates a more focal area [37] and reduces stimulus artifacts [38], the choice of monophasic or biphasic stimulation varies by institution. We used biphasic stimulation in this study since it can more efficiently generate responses, minimize cortical damage, and reduce stimulus artifacts compared to monophasic stimulation [14,16, 27]. Although, SPES studies that do use monophasic stimulation often do so with alternating polarity to reduce charge buildup and polarization of the electrodes and reduce stimulation artifacts in the trial-averaged responses [4,8,13]. While differences in response amplitude with charge, current intensity, or pulse width have been shown using monophasic stimulations [12,13], biphasic stimulations [17–19] or both monophasic and biphasic [16,32], a further study may be needed to explore whether the individual effects of current intensity and pulse width that we observed also apply to monophasic stimulation.

## 6. Conclusion

This study provides a detailed investigation into how current intensity, pulse width, and the resulting charge per phase used in SPES can affect the amplitude, distribution, latency, and morphology of the resulting CCEPs. In the parameter ranges we observed (1.5–7.5 mA and 0.750–1.500 μC), while charge does drive the responses, the individual combination of current intensity and pulse width also affected the results, such that the decreased response metrics at low charges could be mitigated or even reversed by adjusting current intensity and pulse width combinations. Stimulations with a higher current intensity and shorter pulse width resulted in greater response amplitudes and spatial distributions, shorter latency responses, more consistent response waveforms, and lower stimulus artifact amplitudes, sometimes even when the overall stimulus charge was less. This indicates that high current intensities (5 mA and above) and short pulse widths (less than 0.3 ms) may provide the most favorable response metrics, even with a low charge per phase. Our findings may guide the selection of optimal stimulation parameters to maximize the strength and number of elicited CCEPs while reducing stimulus artifacts and minimizing charge.

## Supplementary Material

1

## Figures and Tables

**Table 1:** Pulse width settings used for each current intensity and charge level.

	1.5 mA	2.0 mA	3.0 mA	5.0 mA	7.5 mA
0.750 μC/phase	0.500 ms	0.375 ms	0.250 ms	0.150 ms	0.100 ms
1.125 μC/phase	0.750 ms	0.563 ms	0.375 ms	0.225 ms	0.150 ms
1.500 μC/phase	1.000 ms	0.750 ms	0.500 ms	0.300 ms	0.200 ms

**Table 2:** Patient characteristics.

Patient Number	Sex	Age	Implant Type	Number of Sites Stimulated for Variable Current Intensity and Pulse Width	Total Number of Significant Stimulation-Response Pairs
P1	M	45	SEEG	4	84
P2	F	39	SEEG	3	184
P3	M	20	SEEG and subdural	5	129
P4	F	48	SEEG	3	82
P5	M	33	SEEG	4	86
P6	M	24	SEEG	3	109
P7	F	54	SEEG	4	198
P8	F	19	SEEG	4	189
P9	F	11	SEEG and subdural	5	97
P10	F	48	SEEG	3	139
P11	M	20	Subdural	3	104

M, Male; F, Female; SEEG, Stereoelectroencephalography.

**Table 3:** Response amplitude linear mixed effects model.

Fixed Effect	Coefficient	Standard Error	Degrees of Freedom	t-value	p-value
(Intercept)	2.3191971	0.03378382	18733	68.64817	<0.0001
Current Intensity [mA]	0.0407792	0.00258895	18733	15.75127	<0.0001
Pulse Width [ms]	−0.5819726	0.02520081	18733	−23.09341	<0.0001
Current Intensity * Pulse Width (Charge) [μC]	0.7125032	0.01288317	18733	55.30497	<0.0001

## Abbreviations

CCEP: cortico-cortical evoked potential
EEG: electroencephalography
LRT: likelihood ratio test
RMS: root mean square
SEEG: stereoelectroencephalography
SPES: single pulse electrical stimulation
