# Supplementary material for: Towards optimizing single pulse electrical stimulation: High current intensity, short pulse width stimulation most effectively elicits evoked potentials
Source: Brain Stimul. Author manuscript; Available in PMC 2023 Jul 10. (PMC10330807; doi:10.1016/j.brs.2023.04.023)
Supplement: 1 [file NIHMS1910690-supplement-1.docx]

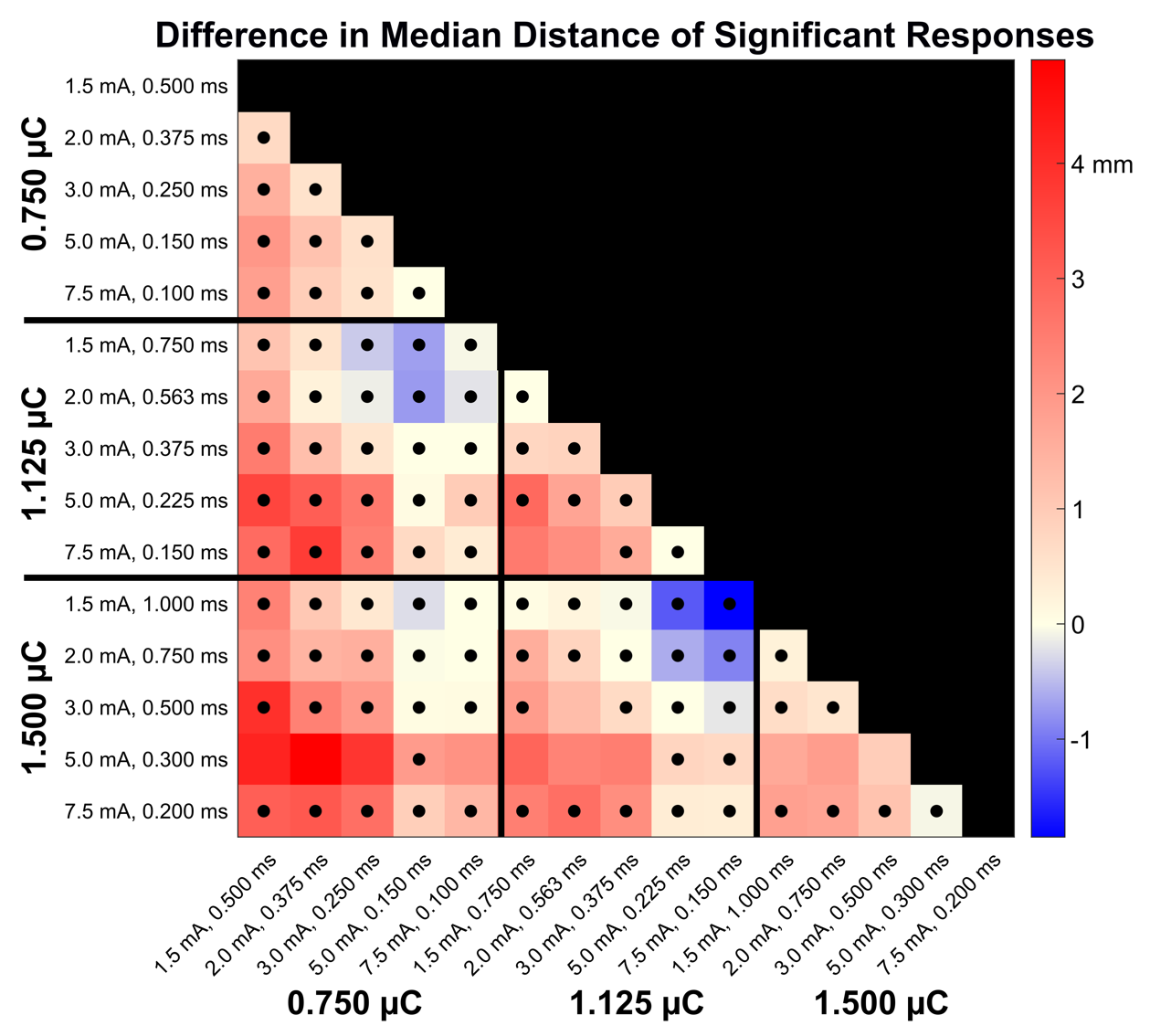


Supplementary Figure 1. Pairwise comparisons of distance of significant responses. The median differences in the median distance from stimulation to each significant response (N1 Z-score > 6) across each stimulation for all possible pairwise comparisons of the 15 stimulation parameter combinations are shown in the matrix. The value of each square represents the condition on the y-axis minus the condition on the x-axis, and the matrix is colored so that a greater value for the condition on the y-axis is colored red and a greater value for the condition on the x-axis is colored blue. Squares marked with a dot represent comparisons with non-significant Wilcoxon signed-rank tests (*P* > 0.05, Bonferroni corrected). Almost every square is dotted here, meaning the differences in distances of significant response across stimulation conditions were largely non-significant.


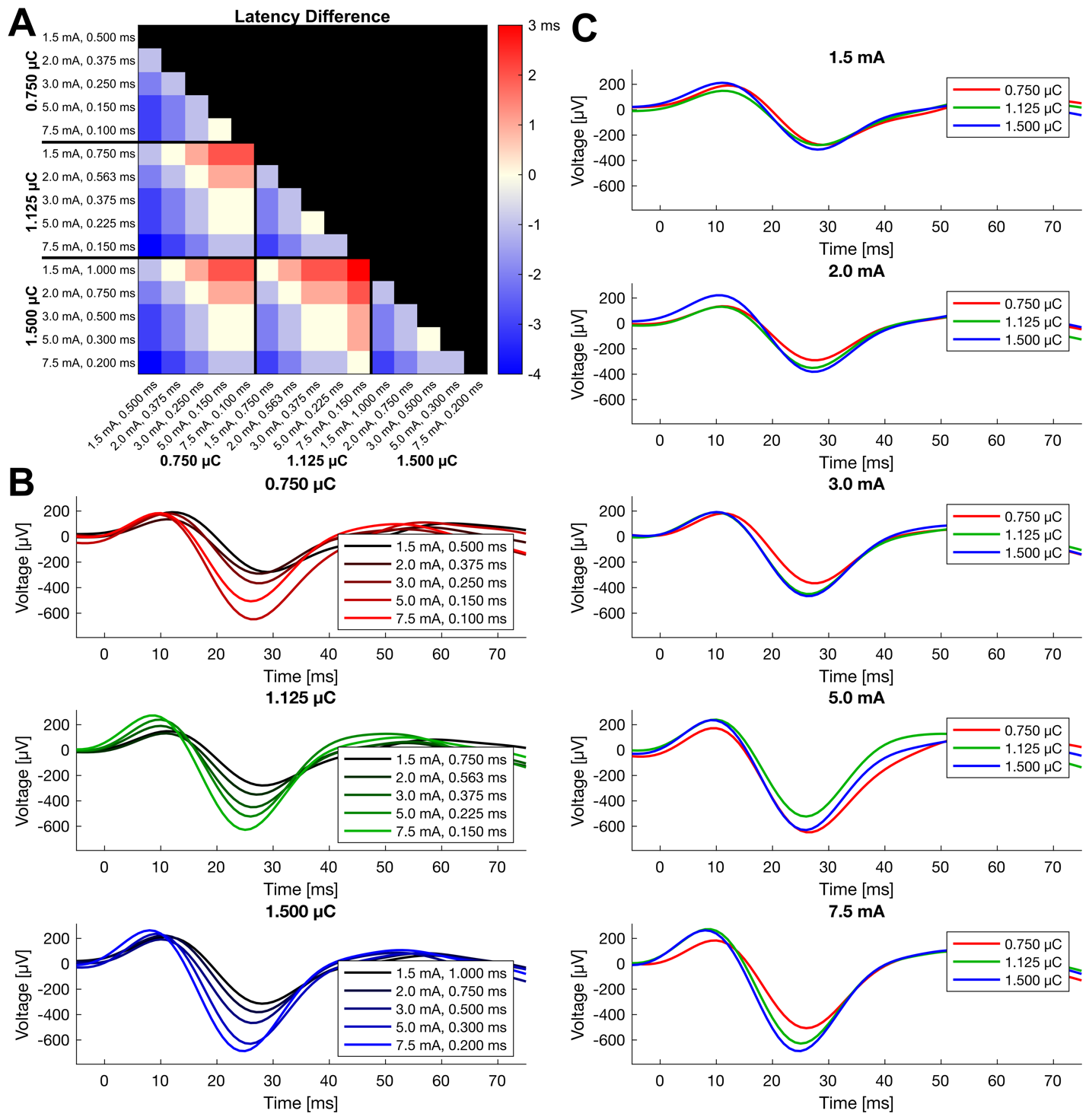


Supplementary Figure 2. Example Effects of Charge, Current Intensity, and Pulse Width on N1 Latency. The responses of a single stimulation-response channel to each stimulation parameter condition are shown as an example visualization of how these parameters can affect the N1 latency. The differences in N1 latency for all possible pairwise comparisons of the 15 stimulation parameter combinations are shown in **A**. The waveforms of the average times series during the N1 time frame in response to each parameter condition are shown grouped by charge (**B**) and current intensity (**C**) to visualize the trends.


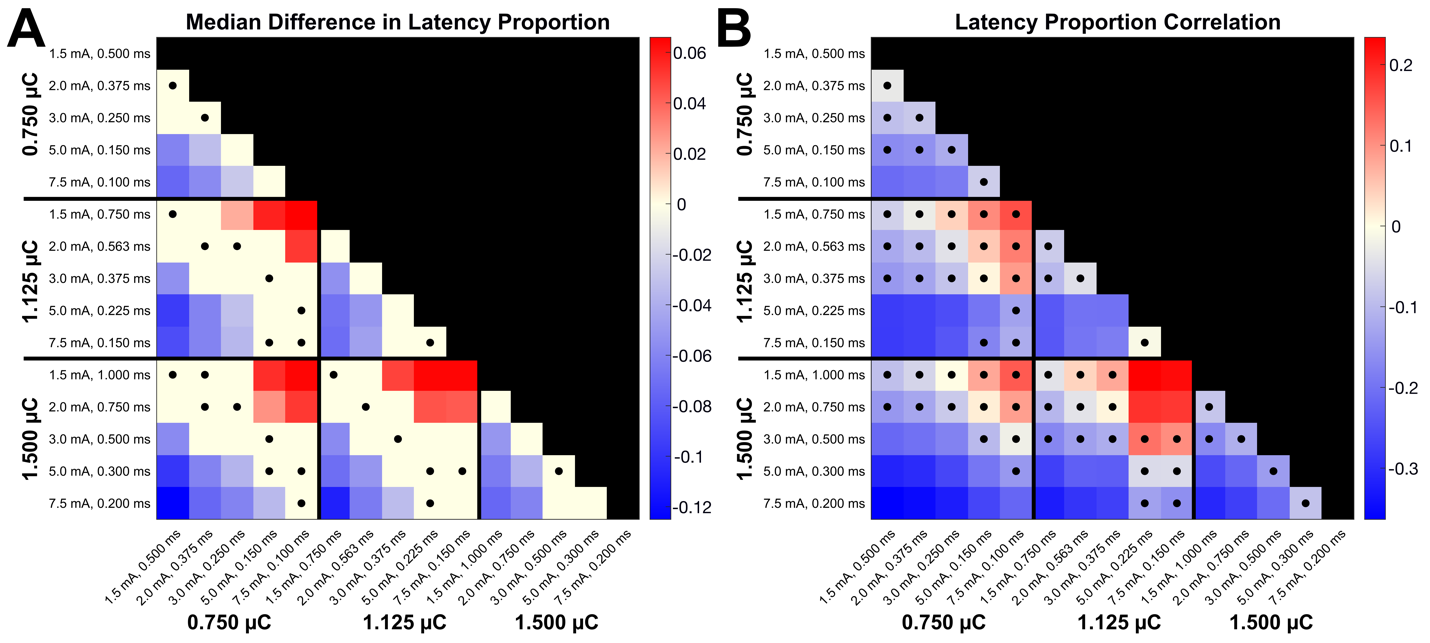


Supplementary Figure 3. Latency Proportion Analysis. The median proportion of the change in N1 response latency with respect to the average N1 latency of that stimulation-response pair across all stimulation-response pairs with significant N1 responses are shown in **A** for all possible pairwise comparisons of the 15 current intensity and pulse width combinations. **B** shows the Pearson correlation between the difference in latency and the average latency across all stimulation-response pairs with significant N1 responses for each pairwise comparison of the stimulation parameters combinations. For each matrix, the value of each square represents the comparison of the condition on the y-axis minus the condition on the x-axis, and the matrix is colored so that a greater value for the condition on the y-axis is colored red and a greater value for the condition on the x-axis is colored blue.


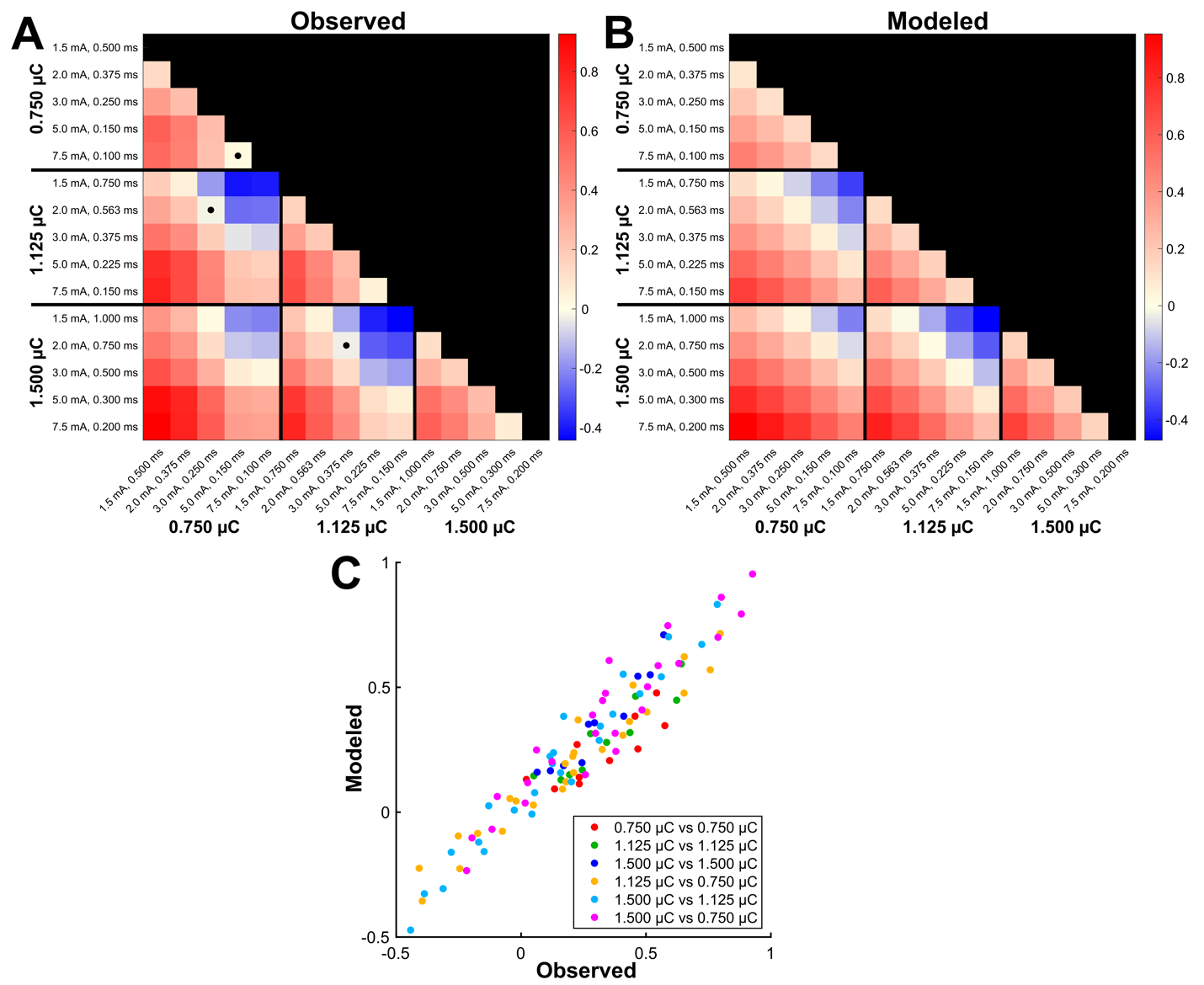


Supplementary Figure 4. Visual comparison of observed and modeled differences in voltage. Observed median differences in voltage across stimulation conditions are shown in **A** (identical to Figure 2C in the main text) and differences in median voltages predicted by the linear mixed effect model with fixed effects of current intensity, pulse width, and charge are shown in **B**. The value of each square represents the condition on the y-axis minus the condition on the x-axis, and the matrix is colored so that a greater value for the condition on the y-axis is colored red and a greater value for the condition on the x-axis is colored blue. The values are dimensionless since they quantify the difference in log-scaled voltages. Squares in C marked with a dot represent comparisons with non-significant Wilcoxon signed-rank tests (*P* > 0.05, Bonferroni corrected). **C**. Scatter plot of the modeled versus observed values shown in **A** and **B**. The Pearson correlation coefficient is 0.95.

Supplementary Table 1. Comparisons of Reduced Linear Mixed Effects Models to Full Model

| Reduced Model | LRT Statistic | df | P Value |
| --- | --- | --- | --- |
| Current Intensity | 3319.185 | 2 | <0.0001 |
| Pulse Width | 6180.003 | 2 | <0.0001 |
| Current Intensity + Pulse Width | 2833.087 | 1 | <0.0001 |
| Charge | 5416.702 | 2 | <0.0001 |
| Current Intensity + Charge | 525.9621 | 1 | <0.0001 |
| Pulse Width + Charge | 246.5225 | 1 | <0.0001 |


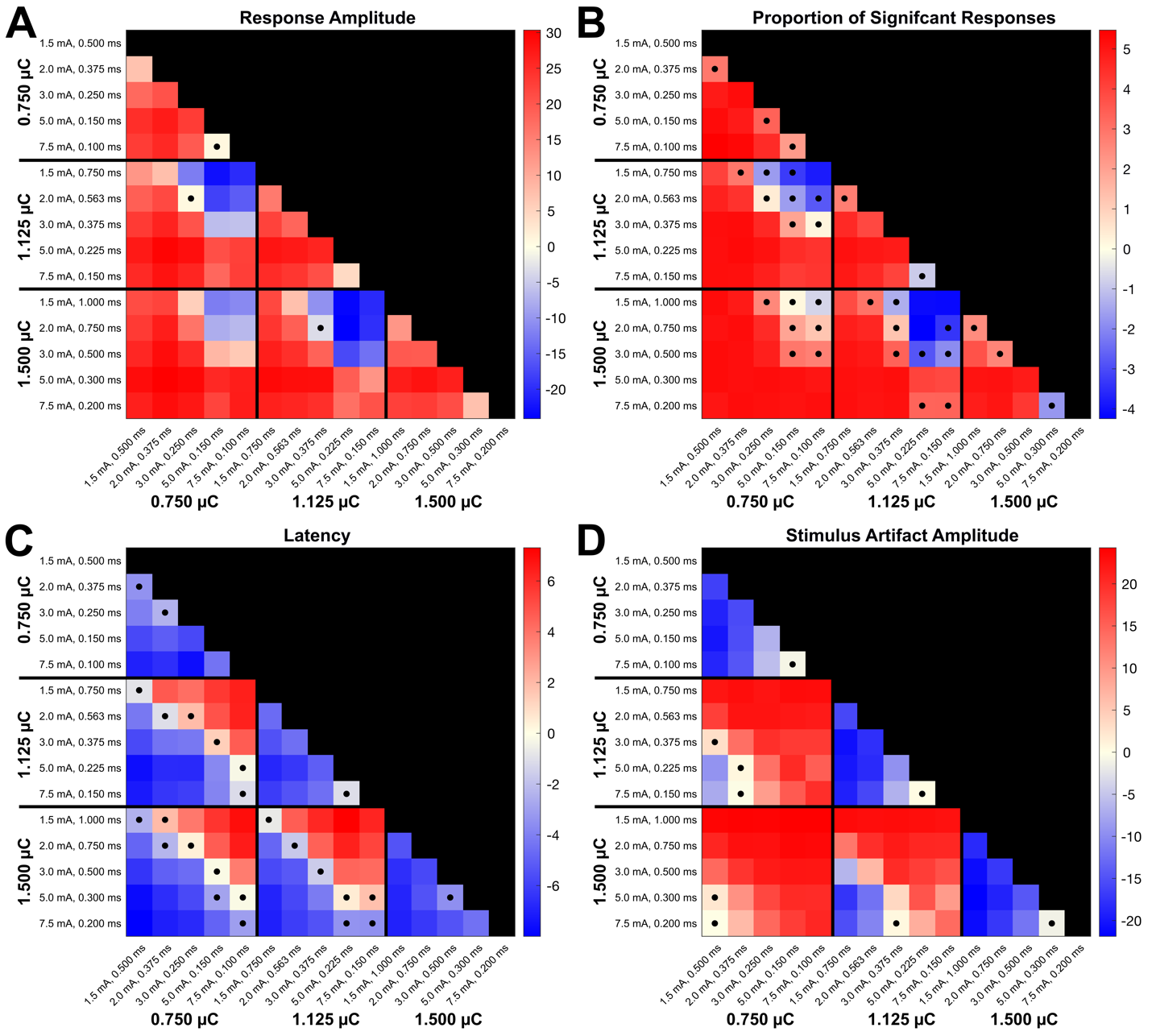


Supplementary Figure 5. Pairwise Wilcoxon signed-rank z-statistics. For each of the pairwise comparisons of response metrics presented in the main text, the z-statistic for the associated Wilcoxon signed-rank test is shown. For each matrix, the value of each square represents the z-statistic for the comparison of the condition on the y-axis minus the condition on the x-axis, and the matrix is colored so that a greater value for the condition on the y-axis is colored red and a greater value for the condition on the x-axis is colored blue.

Supplementary Table 2. Sample Sizes For Wilcoxon Signed-rank Tests

| Metric | Comparisons within 0.750 µC/phase | Comparisons to 1.125 µC/phase and 1.5-3.0 mA at 1.500 µC/phase | Comparisons to 5.0 and 7.5 mA at 1.500 µC/phase |
| --- | --- | --- | --- |
| Response Amplitude | 1424 | 1340 | 1321 |
| Proportion of Significant Responses | 40 | 37 | 36 |
| Latency | 421 | 393 | 391 |
| Stimulus Artifact Amplitude | 879 | 824 | 810 |


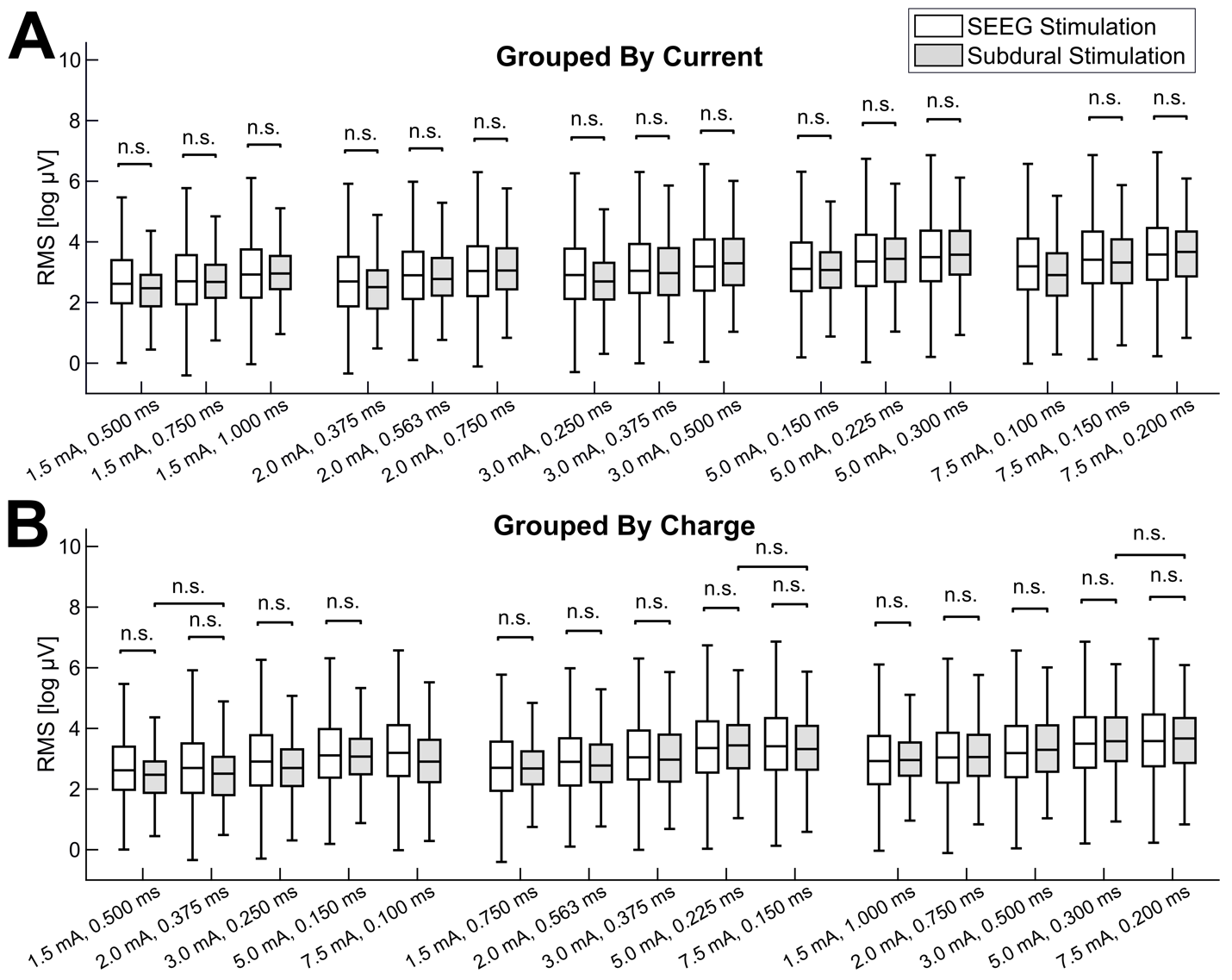


Supplementary Figure 6. Pairwise comparisons of response magnitude between varied current intensity and pulse width combinations separated by SEEG and subdural electrode stimulation. Boxplots of response amplitudes at each parameter combination are shown spatially grouped by current intensity in **A** and spatially grouped by charge in **B**. Wilcoxon signed-rank tests between pairwise comparisons within each current intensity and within each charge level and Wilcoxon rank-sum tests between responses to SEEG and subdural stimulation within each parameter combination were significant (P < 0.05, Bonferroni corrected) except where labeled non-significant (n.s.).
